# Case report: TMEM106B haplotype alters penetrance of GRN mutation in frontotemporal dementia family

**DOI:** 10.3389/fneur.2023.1160248

**Published:** 2023-04-03

**Authors:** Jolien Perneel, Masood Manoochehri, Edward D. Huey, Rosa Rademakers, Jill Goldman

**Affiliations:** ^1^VIB Center for Molecular Neurology, VIB, Antwerp, Belgium; ^2^Department of Biomedical Sciences, University of Antwerp, Antwerp, Belgium; ^3^Department of Neurology, Columbia University, New York, NY, United States; ^4^Department of Neuroscience, Mayo Clinic Jacksonville, Jacksonville, FL, United States

**Keywords:** frontotemporal dementia, progranulin, TMEM106B, disease penetrance, genetic counseling

## Abstract

Frontotemporal dementia (FTD) is the second-most common young-onset dementia. Variants in the *TMEM106B* gene have been proposed as modifiers of FTD disease risk, especially in progranulin (*GRN*) mutation carriers. A patient in their 50s presented to our clinic with behavioral variant FTD (bvFTD). Genetic testing revealed the disease-causing variant c.349 + 1G > C in *GRN*. Family testing revealed that the mutation was inherited from an asymptomatic parent in their 80s and that the sibling also carries the mutation. Genetic analyses showed that the asymptomatic parent and sibling carry two copies of the protective *TMEM106B* haplotype (defined as c.554C > G, p.Thr185Ser), whereas the patient is heterozygous. This case report illustrates that combining *TMEM106B* genotyping with *GRN* mutation screening may provide more appropriate genetic counseling on disease risk in *GRN* families. Both the parent and sibling were counseled to have a significantly reduced risk for symptomatic disease. Implementing *TMEM106B* genotyping may also promote the collection of biosamples for research studies to improve our understanding of the risk-and disease-modifying effect of this important modifier gene.

## 1. Introduction

Frontotemporal dementia (FTD) is the second-most common presenile dementia after Alzheimer’s disease. Up to 40% of affected individuals have a family history of FTD or a related neurodegenerative disease, with 10–30% having an autosomal dominant pattern of inheritance (1). Pathologically, FTD is characterized by the accumulation of Tau, TDP-43 or FUS protein which leads to atrophy of the frontal and temporal regions of the brain referred to as frontotemporal lobar degeneration (FTLD) (2) Mutations in progranulin (*GRN*) account for 5–20% of familial FTLD-TDP and 1–5% of sporadic cases. The phenotype of symptomatic *GRN* carriers has considerable intra-and inter-familial variation, including age of onset, duration of disease, and manifested symptoms. Penetrance is age-related with 90% of carriers being affected by age 70 (3, 4).

**Figure 1:**
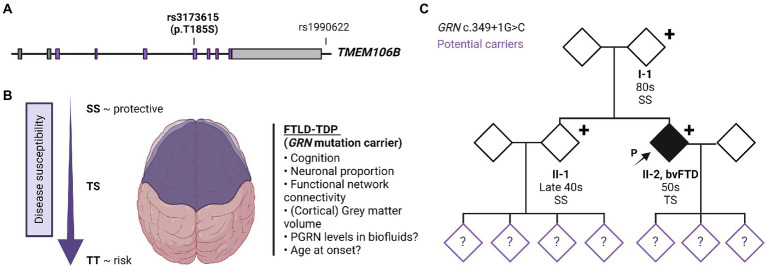
TMEM106B gene structure, *TMEM106B* haplotype-associated phenotypes and family pedigree. **(A)** Gene architecture and effect of risk/protective haplotype of *TMEM106B*. The TMEM106B gene is comprised of nine exons, coding regions are labeled in purple. The major SNPs associated with neurodegenerative diseases are indicated with sentinel SNP (rs1990622), located in the regulatory sequence downstream of *TMEM106B*, and coding variant rs3173615 (c.554C > G, p.Thr185Ser). **(B)** Current understanding of the effect of *TMEM106B* haplotype on brain health and disease susceptibility in FTLD-*GRN*. **(C)** Family pedigree. Patient II-2 presented in the clinic in their 50s with symptoms of bvFTD. Genetic testing revealed a disease-causing variant in *GRN* (c.349 + 1G > C, Splice donor). The patient’s asymptomatic parent (I-1) and younger sibling (II-1) who also carry the mutation were evaluated in their 80s and late 40s, respectively. *TMEM106B* genotyping showed I-1 and II-1 to have two copies of the protective *TMEM106B* allele (SS), while the patient is heterozygous (TS). The children of II-1 and II-2, were not genetically tested but are at risk of carrying the mutation, as represented with a question mark within the symbol. Figure was created using BioRender.com.

*TMEM106B* was first identified as a risk-associated gene for FTD with TDP-43 pathology (FTLD-TDP) and since then has been recognized as an important modifier of disease risk in a variety of neurodegenerative disorders [reviewed in (5, 6)]. Multiple single nucleotide polymorphisms (SNPs) are present at the *TMEM106B* locus on chromosome 7p21 which are in strong linkage disequilibrium (LD). Consequently, it has been difficult to pinpoint the specific functional variant (s) modulating the disease risk. The genetic status of *TMEM106B* is therefore usually described as either a risk or protective haplotype with the most significant SNP from the original genome-wide association study, rs1990622, often stated as the sentinel SNP representing the haplotype (Figure 1A). Here, the major T allele is associated with an increased risk and the minor C allele with a reduced risk for developing disease (7). Alternatively, the two haplotypes can be differentiated by the only coding variant rs3173615 (c.554C > G) where the risk haplotype carries a Threonine and the protective haplotype carries a Serine at position 185.

While the functional variant remains a topic of active discussion, the *TMEM106B* haplotype is proposed to alter TMEM106B expression, with an increased expression correlating with the risk haplotype. The non-coding variant, rs1990620, is proposed to modulate TMEM106B expression through transcriptional activation due to altered long-range chromatin-looping interactions (8) while the one coding variant (rs3173615, p.T185S), located in TMEM106B’s fourth N-X-T/S glycosylation motif, may affect TMEM106B protein levels by affecting the protein stability and degradation rate due to differences in N-glycosylation (9). As an integral lysosomal transmembrane protein, TMEM106B regulates several aspects of lysosomal functioning, and proper TMEM106B protein levels are crucial for maintaining lysosomal health.

Interestingly, the risk-modifying effect of *TMEM106B* is most prominent in FTLD-TDP patients harboring disease-associated *GRN* mutations (4) and the premise that the alteration in TMEM106B levels is the driver of the disease-modulating effect is further substantiated by the observation that TMEM106B mRNA and protein levels were significantly increased in *GRN* mutation carriers (10, 11). Considering, PGRN is cleaved within the lysosome into functional granulins and also affects several aspects of lysosomal function, it is likely that the exceptionally strong disease-modifying effect in *GRN* mutation carriers occurs within the endolysosomal system, but the precise mechanism remains unknown.

Functionally, the *TMEM106B* ‘risk’ haplotype has been associated with lower progranulin levels (12, 13), reduced volume of the superior temporal gyrus (especially in the left hemisphere) (14), decreased functional network connectivity (15), decreased neuronal proportion (16), and a faster cognitive decline (17) (Figure 1B). The association with cognition, neuronal proportion, and general brain health was also replicated in the absence of brain disease (18), suggesting that *TMEM106B* may modulate the susceptibility of an individual to the pathophysiology of FTLD and related disorders and functions as natural protection against neurodegeneration in general. However, despite the clear functional effect of the *TMEM106B* haplotype, *TMEM106B* genotyping is not routinely implemented in diagnostic testing.

## 2. Case description

### 2.1. Clinical presentation

**Figure 2:**
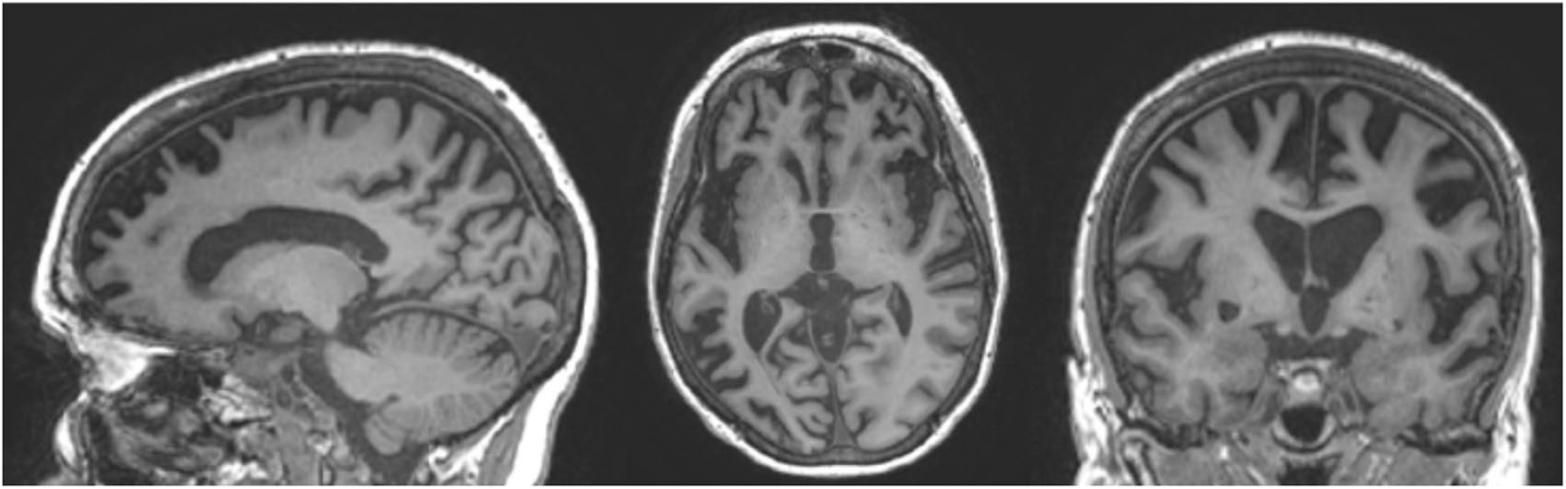
MRI images of the proband at evaluation. Portrayed are representative images from a T1-weighted sequence indicating severe global atrophy, slightly right > left, most pronounced in frontal, temporal, and parietal regions, and deemed consistent with behavioral variant frontotemporal dementia.

A patient in their 50s presented to our clinic with progressive cognitive and behavioral impairment (Patient II-2; Figure 1C). The patient’s partner reported that the proband suffered from cognitive changes starting a year earlier with forgetfulness, difficulty with calculations and computers, impaired judgment while driving, behavioral changes, and lack of initiative in household chores. Concurrent with onset of cognitive symptoms, the patient had prominent dietary changes, including increased appetite and sweet cravings, resulting in a 30 pounds weight gain. Mood swings including episodes of crying were also noted, for which the patient was treated with Zoloft with some benefit. Psychotic symptoms included visual illusions, paranoia, and rare auditory hallucinations. The patient had partial insight into their cognitive and behavioral changes, with relative preservation of emotional range such as maintained interest in their family. The clinical impression was behavioral variant frontotemporal dementia (bvFTD), which the neuropsychological testing and neuroimaging workup supported (Table 1; Figure 2).

**Table 1:** Family member information and results of neurological assessment.

ID	II-2	II-1	I-1
Disease-causing *GRN* variant (c.349 + 1G > C)	Heterozygous	Heterozygous	Heterozygous
*TMEM106B* variant (c.554C > G, p.Thr185Ser)	Heterozygous (TS)	Homozygous (SS)	Homozygous (SS)
Age at assessment	50s	Late 40s	80s
Education	16	19	14
Neurological exam	Within normal limits	Brisk reflexes but within normal limits	Within normal limits
Neuropsychological profile^*^			N/A
Orientation	Within normal limits	Within normal limits	–
Attention	Below normal limits	Within normal limits	–
Visuospatial	Below normal limits	Within normal limits	–
Executive Function	Below normal limits	Variable	–
Processing Speed	Below normal limits	Variable	–
Language	Below normal limits	Variable	–
Verbal Memory (Learning)	Variable	Within normal limits	–
Verbal Memory (Retention)	Within normal limits	Within normal limits	–
Working Memory	Below normal limits	Within normal limits	–
MRI	Severe global atrophy, slightly right > left, most pronounced in frontal, temporal, and parietal regions	Within normal limits	N/A
FDG-PET	Severe bilateral frontal and right temporal, moderate–severe right parietal, and moderate left parietal hypometabolism	N/A	N/A

* Note on neuropsychology labels: “Within normal limits” indicates scores on tests > 24%ile, “Below normal limits” indicates scores on tests ≤ 24%ile, “Variable” indicates inconsistent test performance within the domain, but overall intact.

### 2.2. Diagnostic screening and family work-up

The patient’s family history was unremarkable (Figure 1C). The parents are still living (in their 80s) without any neurological or psychological symptoms. The patient has a healthy sibling (in their late 40s) and three healthy adult children. Much of the family history was lost due to grandparents and many other family members dying during the World War II era. Despite a lack of family history, we performed a full dementia-ALS genetic testing panel as well as expansion testing for the hexanucleotide repeat in *C9orf72* in patient II-2. The only disease-causing variant identified was a previously reported variant, c.349 + 1G > C (Splice donor site), in *GRN* (19) This variant is predicted to lead to skipping of exon 3, introducing a premature termination codon, nonsense-mediated decay, and loss of progranulin protein.

In order to understand the origin of this mutation, the patient’s parents and sibling were also tested which revealed that both the parent (I-1) and sibling (II-1) also carried the mutation. We subsequently evaluated the patient’s parent (I-1) which revealed no current neuropsychiatric or behavioral symptoms. The psychiatric history was also unremarkable. The parent had no reported difficulties performing ADLs and IADLs and brief cognitive testing on the Mini Mental State Exam revealed intact cognition. On video examination, the parent was socially appropriate and there were no motor abnormalities or Parkinsonism. The patient’s sibling (II-1) was also evaluated, which revealed no significant cognitive changes, socially inappropriate behavior, or other symptoms consistent with bvFTD. The sibling did endorse some longstanding navigational problems as well as more recent subjective memory complaints, low mood, and anxiety. These recent changes were judged to be likely related to their concern about the patient’s condition and the recent familial genetic findings. Neuropsychological testing was largely within normal limits (Table 1). Neurological examination was normal, with the exception of brisk reflexes.

### 2.3. Genetic analyses of *TMEM106B*

The family wished to understand the reason why the parent was asymptomatic despite carrying the same mutation. Since we previously identified *TMEM106B* as the only genome-wide significant modifier of disease risk in *GRN* mutation carriers using a population of symptomatic individuals, *TMEM106B* genetic testing was performed in all *GRN* mutation carriers from the family. This analysis revealed that the asymptomatic parent and sibling both carry two copies of the protective *TMEM106B* haplotype (rs3173615; SS at position 185) while the patient was found to be heterozygous (rs3173615, ST at position 185). Based on our previous study (4) the family was counseled that the parent and sibling have a theoretical 50% reduced risk to develop symptoms as compared to those with no protective haplotypes.

## 3. Conclusion

We present a *GRN* family with a proband presenting with classical young-onset FTD whose parent is an asymptomatic carrier in their 80s, possibly protected from developing disease symptoms because of the modifying effect of *TMEM106B*. This report is in line with our previously reported *TMEM106B* genetic study in unrelated *GRN* patients, in which very few of the symptomatic *GRN* carriers were homozygous for the *TMEM106B* protective haplotype (4), and suggests that the presence of at least one *TMEM106B* risk haplotype is required - as a permissive haplotype - to develop FTLD-*GRN*. This family report highlights the importance of genotyping FTD patients and relatives for their respective *TMEM106B* haplotypes, especially in at-risk *GRN* mutation carriers.

Given the diverse and repeatedly demonstrated modifying effect of *TMEM106B* on the development and presentation of neurodegenerative brain diseases, it is surprising that *TMEM106B* genotyping is usually not reported in scientific publications and is also not routinely implemented in diagnostic profiling. Even though the specific functional variant(s) responsible for the disease-modifying effect is not known, candidate functional variants are in strong LD (rs1990622 and rs3173615; *r*^2^ = 0.976 in European populations), and genotyping either one of the variants will provide the necessary information. We argue that *TMEM106B* genetic testing for the ‘risk haplotype’ could be established by sequencing for the rs3173615 variant as it is the only coding variant differentiating the permissive (T) from the protective (S) haplotype and therefore present in exome sequencing data, which is the predominant sequencing method used for genetic testing.

The lack of systematic genotyping of *TMEM106B* in *GRN* families has hampered identification of *GRN* carriers who are also homozygous for the *TMEM106B* ‘protective’ haplotype (and thus often remain without symptoms) simply because they do not show up in the clinic to participate in research studies. Moreover, the few *GRN* carriers who were reported to develop symptoms without carrying a *TMEM106B* risk haplotype may have developed disease due to additional risk factors or advanced aging or have in fact a different disease etiology. Detailed investigation of these individuals will be necessary as it is possible that the molecular mechanism underlying disease in *GRN* carriers that do not carry the permissive risk haplotype is mechanistically distinct; information that will be vital, especially in light of the development of gene-based therapies for *GRN* mutation carriers.

In sum, diagnostic testing for the *TMEM106B* haplotype does not only hold potential to improve genetic counseling in *GRN* families but will also facilitate further studies by enabling the collection of patient data and material (such as biofluids) crucial for research. A deeper understanding of the risk- and disease-modifying effect of TMEM106B will be essential and could hold the key towards new insights and therapeutic avenues for FTD and related neurodegenerative diseases.

## 4. Methods

### 4.1. Patient consent and ethical approval

Data from the proband, the parent, and sibling were obtained through research overseen and approved by the Institutional Review Boards at the Johns Hopkins School of Medicine and Columbia University Irving Medical Center; (JHM IRB00227492, CUIMC IRB-AAAS8862, CUIMC IRB-AAAP1303). The patient and family provided approval to share information in this case report and the family pedigree was anonymized. The changes do not affect the current description and conclusion of this report.

### 4.2. Clinical evaluation

Clinical evaluations of the proband, the parent, and sibling were completed at Columbia University Irving Medical Center as part of their involvement in the following NIA-sponsored research studies: ARTFL-LEFFTDS Longitudinal Frontotemporal Lobar Degeneration (ALLFTD) and Neuroanatomical associations with the factor structure underlying neuropsychiatric symptoms in Alzheimer’s disease (NAPS).

### 4.3. Genetic analyses

Saliva samples from the proband, the parents, and sibling were collected and sent to Invitae Laboratory, San Francisco, California for genetic testing. A 33-gene Hereditary Amyotrophic Lateral Sclerosis, Frontotemporal Dementia, and Alzheimer’s Disease Panel was analyzed by next-generation sequencing followed by analysis of *GRN* (c.349 + 1G > C) and *TMEM106B* (c.554C > G).

## Data availability statement

The datasets presented in this article are not readily available because of ethical and privacy restrictions. Requests to access the datasets should be directed to the corresponding author.

## Ethics statement

The studies involving human participants were reviewed and approved by Institutional Review Boards at the Johns Hopkins School of Medicine and Columbia University. The patients/participants provided their written informed consent to participate in this study. Written informed consent was obtained for the publication of this case report.

## Author contributions

EH and JG neurologically evaluated and counseled the proband and family. MM completed research testing with the proband and family. EH, JG, and MM helped to critically review and revise the manuscript. RR and JP provided insight into the interaction between progranulin and TMEM106B and drafted the manuscript. All authors made substantial contributions to the discussion of the content, reviewed, and edited the article.

## Funding

The work was supported by the University of Antwerp Research Funds (BOF) and Vlaams Instituut voor Biotechnologie (VIB), as well as the National Institutes of Health (NIH) grants AG062268, UG3NS103870 and the ARTFL LEFFTDS Longitudinal Frontotemporal Lobar Degeneration grant (ALLFTD; U19AG063911). PJ is supported by a fellowship from Research Foundation—Flanders (FWO, application 11M1622N).

## Conflict of interest

RR is a member of the Scientific Advisory Board of Arkuda Therapeutics and receives invention royalties from a patent related to progranulin.

The remaining authors declare that the research was conducted in the absence of any commercial or financial relationships that could be construed as a potential conflict of interest.

## Publisher’s note

All claims expressed in this article are solely those of the authors and do not necessarily represent those of their affiliated organizations, or those of the publisher, the editors and the reviewers. Any product that may be evaluated in this article, or claim that may be made by its manufacturer, is not guaranteed or endorsed by the publisher.

## References

[1] Goldman JS van Deerlin VM. Alzheimer’s disease and frontotemporal dementia: the current state of genetics and genetic testing since the advent of next-generation sequencing. Mol Diagn Ther. (2018) 22:505–13. doi: 10.1007/s40291-018-0347-7, PMID: 29971646 PMC6472481

[2] Olney NT Spina S Miller BL. Frontotemporal dementia. Neurol Clin. (2017) 35:339–74. doi: 10.1016/J.NCL.2017.01.008, PMID: 28410663 PMC5472209

[3] Rademakers R Neumann M MacKenzie IR. Advances in understanding the molecular basis of frontotemporal dementia. Nat Rev Neurol. (2012) 8:423–34. doi: 10.1038/nrneurol.2012.117, PMID: 22732773 PMC3629543

[4] Pottier C Zhou X Perkerson RB Baker M Jenkins GD Serie DJ. Potential genetic modifiers of disease risk and age at onset in patients with frontotemporal dementia and GRN mutations: a genome-wide association study. Lancet Neurol. (2018) 17:548–58. doi: 10.1016/S1474-4422(18)30126-1, PMID: 29724592 PMC6237181

[5] Nicholson AM Rademakers R. What we know about TMEM106B in neurodegeneration. Acta Neuropathol. (2016) 132:639–51. doi: 10.1007/S00401-016-1610-9, PMID: 27543298 PMC5074873

[6] Feng T Lacrampe A Hu F. Physiological and pathological functions of TMEM106B: a gene associated with brain aging and multiple brain disorders. Acta Neuropathol. (2021) 141:327–39. doi: 10.1007/S00401-020-02246-3, PMID: 33386471 PMC8049516

[7] van Deerlin VM Sleiman PMA Martinez-Lage M Chen-Plotkin A Wang LS Graff-Radford NR. Common variants at 7p21 are associated with frontotemporal lobar degeneration with TDP-43 inclusions. Nat Genet. (2010) 42:234–9. doi: 10.1038/NG.536, PMID: 20154673 PMC2828525

[8] Gallagher MD Posavi M Huang P Unger TL Berlyand Y Gruenewald AL. A dementia-associated risk variant near TMEM106B alters chromatin architecture and gene expression. Am J Hum Genet. (2017) 101:643–63. doi: 10.1016/j.ajhg.2017.09.004, PMID: 29056226 PMC5673619

[9] Nicholson AM Finch NA Wojtas A Baker MC Perkerson RB Castanedes-Casey M. TMEM106B p.T185S regulates TMEM106B protein levels: implications for frontotemporal dementia. J Neurochem. (2013) 126:781–91. doi: 10.1111/jnc.12329, PMID: 23742080 PMC3766501

[10] Chen-Plotkin AS Unger TL Gallagher MD Bill E Kwong LK Volpicelli-Daley L. TMEM106B, the risk gene for frontotemporal dementia, is regulated by the microRNA-132/212 cluster and affects progranulin pathways. J Neurosci. (2012) 32:11213–27. doi: 10.1523/JNEUROSCI.0521-12.2012, PMID: 22895706 PMC3446826

[11] Busch JI Martinez-Lage M Ashbridge E Grossman M van Deerlin VM Hu F. Expression of TMEM106B, the frontotemporal lobar degeneration-associated protein, in normal and diseased human brain. Acta Neuropathol Commun. (2014) 1:36. doi: 10.1186/2051-5960-1-36, PMID: 24252750 PMC3893524

[12] Cruchaga C Graff C Chiang HH Wang J Hinrichs AL Spiegel N. Association of TMEM106B gene polymorphism with age at onset in granulin mutation carriers and plasma granulin protein levels. Arch Neurol. (2011) 68:581–6. doi: 10.1001/ARCHNEUROL.2010.350, PMID: 21220649 PMC3090529

[13] Finch N Carrasquillo MM Baker M Rutherford NJ Coppola G Dejesus-Hernandez M. TMEM106B regulates progranulin levels and the penetrance of FTLD in GRN mutation carriers. Neurology. (2011) 76:467–74. doi: 10.1212/WNL.0b013e31820a0e3b, PMID: 21178100 PMC3034409

[14] Adams HHH Verhaaren BFJ Vrooman HA Uitterlinden AG Hofman A van Duijn CM. TMEM106B influences volume of left-sided temporal lobe and interhemispheric structures in the general population. Biol Psychiatry. (2014) 76:503–8. doi: 10.1016/j.biopsych.2014.03.006, PMID: 24731779

[15] Premi E Formenti A Gazzina S Archetti S Gasparotti R Padovani A. Effect of TMEM106B polymorphism on functional network connectivity in asymptomatic GRN mutation carriers. JAMA Neurol. (2014) 71:216–21. doi: 10.1001/jamaneurol.2013.4835 24343233

[16] Li Z Farias FHG Dube U Del-Aguila JL Mihindukulasuriya KA Fernandez MV. The TMEM106B FTLD-protective variant, rs 1990621, is also associated with increased neuronal proportion. Acta Neuropathol. (2020) 139:45–61. doi: 10.1007/s00401-019-02066-0, PMID: 31456032 PMC6942643

[17] Tropea TF Mak J Guo MH Xie SX Suh E Rick J. TMEM106B effect on cognition in Parkinson disease and frontotemporal dementia. Ann Neurol. (2019) 85:801–11. doi: 10.1002/ana.25486, PMID: 30973966 PMC6953172

[18] Rhinn H Abeliovich A. Differential aging analysis in human cerebral cortex identifies variants in TMEM106B and GRN that regulate aging phenotypes. Cell Syst. (2017) 4:404–415.e5. doi: 10.1016/j.cels.2017.02.009, PMID: 28330615

[19] Moore KM Nicholas J Grossman M McMillan CT Irwin DJ Massimo L. Age at symptom onset and death and disease duration in genetic frontotemporal dementia: an international retrospective cohort study. Lancet Neurol. (2020) 19:145–56. doi: 10.1016/S1474-4422(19)30394-1, PMID: 31810826 PMC7007771

